# 1-(2,3,4,6-Tetra-*O*-acetyl-β-d-gluco­pyranos­yl)-3-thio­ureidothio­urea monohydrate

**DOI:** 10.1107/S1600536808043833

**Published:** 2009-01-08

**Authors:** Weidong Sun, Jin Yao, Lifei Bai, Xiaoming Wang

**Affiliations:** a Department of Chemistry, Chifeng College, Chifeng 024001, People’s Republic of China; b College of Chemistry, Nanjing University, Hankou Road, Nanjing,210093, People’s Republic of China; c Jiangsu Key Laboratory of Chinese Medicine Processing, Nanjing University of Chinese Medicine, Nanjing, 210029, People’s Republic of China; d State Key Laboratory of Pharmaceutical Biotechnology, School of Life Science, Nanjing University, Hankou Road, Nanjing,210093, People’s Republic of China

## Abstract

In the title compound, C_16_H_24_N_4_O_9_S_2_·H_2_O, the hexopyranosyl ring adopts a chair conformation (^4^ *C*_1_), and the five substituents are in equatorial positions. In the crystal structure, extensive O—H⋯O, N—H⋯S and N—H⋯O hydrogen bonding leads to the formation of a three-dimensional network.

## Related literature

For cyclo­addition and nucleophilic addition, see: Pearson *et al.* (2003 ▶); Reitz *et al.* (1989 ▶). For the crystal structure of glycosyl isothio­syanate, see: Jiang *et al.* (2003 ▶). For the crystal structures of glycosyl isothio­syanate methanol and ethanol derivatives, see: Zhang *et al.* (2001 ▶).
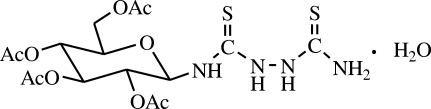


## Experimental

### 

#### Crystal data

C_16_H_24_N_4_O_9_S_2_·H_2_O
*M* *_r_* = 498.53
Monoclinic, 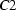

*a* = 22.286 (2) Å
*b* = 7.2005 (7) Å
*c* = 15.8772 (17) Å
β = 110.119 (2)°
*V* = 2392.3 (4) Å^3^
*Z* = 4
Mo *K*α radiation
μ = 0.28 mm^−1^
*T* = 293 (2) K
0.45 × 0.22 × 0.22 mm

#### Data collection

Bruker SMART CCD area-detector diffractometer
Absorption correction: none
6322 measured reflections
3525 independent reflections
3021 reflections with *I* > 2σ(*I*)
*R*_int_ = 0.036

#### Refinement

*R*[*F* ^2^ > 2σ(*F* ^2^)] = 0.055
*wR*(*F* ^2^) = 0.141
*S* = 1.07
3525 reflections
289 parameters
7 restraints
H-atom parameters constrained
Δρ_max_ = 0.42 e Å^−3^
Δρ_min_ = −0.27 e Å^−3^
Absolute structure: Flack (1983 ▶), 1229 Friedel pairs
Flack parameter: −0.16 (12)

### 

Data collection: *SMART* (Bruker, 2007 ▶); cell refinement: *SAINT* (Bruker, 2007 ▶); data reduction: *SAINT*; program(s) used to solve structure: *SHELXS97* (Sheldrick, 2008 ▶); program(s) used to refine structure: *SHELXL97* (Sheldrick, 2008 ▶); molecular graphics: *SHELXTL* (Sheldrick, 2008 ▶); software used to prepare material for publication: *SHELXTL*.

## Supplementary Material

Crystal structure: contains datablocks global, I. DOI: 10.1107/S1600536808043833/su2088sup1.cif

Structure factors: contains datablocks I. DOI: 10.1107/S1600536808043833/su2088Isup2.hkl

Additional supplementary materials:  crystallographic information; 3D view; checkCIF report

## Figures and Tables

**Table 1:** Hydrogen-bond geometry (Å, °)

*D*—H⋯*A*	*D*—H	H⋯*A*	*D*⋯*A*	*D*—H⋯*A*
O1*W*—H10⋯O5^i^	0.87	2.64	3.382 (11)	146
O1*W*—H20⋯O9^ii^	0.87	2.56	3.181 (9)	129
N1—H1*A*⋯S2^iii^	0.86	2.62	3.400 (4)	151
N2—H2*A*⋯O3^iv^	0.86	2.09	2.856 (5)	147
N3—H3*A*⋯O1*W*^v^	0.86	2.13	2.973 (9)	167
N4—H4*B*⋯O1*W*^vi^	0.86	2.43	3.244 (9)	159
N4—H4*C*⋯O1^iii^	0.86	2.49	3.323 (5)	164

Symmetry codes: (i) 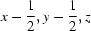; (ii) 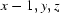; (iii) 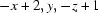; (iv) 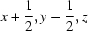; (v) 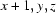; (vi) 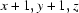.

**Fig. 1.**
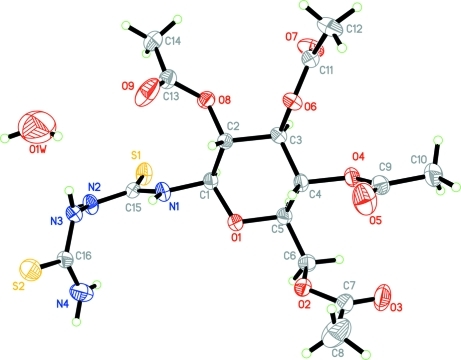
A view of the molecular structure of compound (I), showing the atom-labelling scheme and displacement ellipsoids drawn at the 50% probability level.

## supplementary crystallographic information

### Comment

Over the past decade, many organic chemists have been engaged in the synthesis of glycosyl isothiosyanates and its derivatives. These compound are versatile reagents in organic synthesis and easily undergo many important reactions, such as cycloaddition (Pearson *et al.*, 2003) and nucleophilic addition (Reitz *et al.*, 1989). Recently, the crystal structures of glycosyl isothiosyanate (Jiang *et al.*, 2003) and the methanol and ethanol derivatives (Zhang *et al.*, 2001) have been reported. However, other derivatives of glycosyl isothiosyanate are still rare. Here we report on the synthesis of a new thiosemicarbazide derivative of glycosyl isothiosyanate, 2,3,4,6-tetra-*O*-acetyl- β-*D*-glucopyranosyl dithiourea, (I).

The molecular structure of compound (I) is illustrated in Fig. 1. The hexopyranosyl ring adopts a chair conformation (^4^C_1_), and the four substituents are in equatorial positions.

In the crystal extensive O—H···O, N—H···S and N—H···O hydrogen bonding (Table 1) leads to the formation of a three-dimensional network.

### Experimental

Compound (I) was prepared by refluxing together equimolar amounts of β-*D*-2,3,4,6-tetra-*O*- acetyl-glucopyranosyl isothiocyanate and thiosemicarbazide. After cooling to room temperature, water was added to the mixture and compound (I) was isolated as a white solid. Crystals, suitable for X-ray analysis, were grown from an ethyl acetate and acetonitrile (1:1 / v:v) solution by slow evaporation at room temperature.

### Refinement

The compound has a known chiral center [the Flack parameter is -0.16 (12) (Flack, 1983)], and for this reason the Friedel pairs were not merged. The water H-atoms were located in the difference Fourier maps and refined with distance restraintes, O-H = 0.87 (2) Å. The N- and C-bound H-atoms were placed in calculated positions and treated as riding atoms: N—H = 0.86 Å, C—H = 0.96 - 0.98 Å, with U_iso_(H) = 1.2 or 1.5U_eq_(parent N- or C-atom).

### Crystal data

**Table:**

C_16_H_24_N_4_O_9_S_2_·H_2_O	*F*(000) = 1048
*M**_r_* = 498.53	*D*_x_ = 1.384 Mg m^−^^3^
Monoclinic, *C*2	Melting point: not measured K
Hall symbol: C 2y	Mo *K*α radiation, λ = 0.71073 Å
*a* = 22.286 (2) Å	Cell parameters from 7141 reflections
*b* = 7.2005 (7) Å	θ = 1.4–27.7°
*c* = 15.8772 (17) Å	µ = 0.28 mm^−^^1^
β = 110.119 (2)°	*T* = 293 K
*V* = 2392.3 (4) Å^3^	Block, colorless
*Z* = 4	0.45 × 0.22 × 0.22 mm

### Data collection

**Table:**

Bruker SMART CCD area-detector diffractometer	3021 reflections with *I* > 2σ(*I*)
Radiation source: fine-focus sealed tube	*R*_int_ = 0.036
graphite	θ_max_ = 25.0°, θ_min_ = 1.4°
φ scans, and ω scans	*h* = −25→26
6322 measured reflections	*k* = −8→8
3525 independent reflections	*l* = −18→11

### Refinement

**Table:**

Refinement on *F*^2^	Secondary atom site location: difference Fourier map
Least-squares matrix: full	Hydrogen site location: inferred from neighbouring sites
*R*[*F*^2^ > 2σ(*F*^2^)] = 0.055	H-atom parameters constrained
*wR*(*F*^2^) = 0.141	*w* = 1/[σ^2^(*F*_o_^2^) + (0.0808*P*)^2^] where *P* = (*F*_o_^2^ + 2*F*_c_^2^)/3
*S* = 1.07	(Δ/σ)_max_ < 0.001
3525 reflections	Δρ_max_ = 0.42 e Å^−^^3^
289 parameters	Δρ_min_ = −0.27 e Å^−^^3^
7 restraints	Absolute structure: Flack (1983), 1229 Friedel pairs
Primary atom site location: structure-invariant direct methods	Flack parameter: −0.16 (12)

### Special details

**Table:**

Geometry. All e.s.d.'s (except the e.s.d. in the dihedral angle between two l.s. planes) are estimated using the full covariance matrix. The cell e.s.d.'s are taken into account individually in the estimation of e.s.d.'s in distances, angles and torsion angles; correlations between e.s.d.'s in cell parameters are only used when they are defined by crystal symmetry. An approximate (isotropic) treatment of cell e.s.d.'s is used for estimating e.s.d.'s involving l.s. planes.
Refinement. Refinement of *F*^2^ against ALL reflections. The weighted *R*-factor *wR* and goodness of fit *S* are based on *F*^2^, conventional *R*-factors *R* are based on *F*, with *F* set to zero for negative *F*^2^. The threshold expression of *F*^2^ > σ(*F*^2^) is used only for calculating *R*-factors(gt) *etc*. and is not relevant to the choice of reflections for refinement. *R*-factors based on *F*^2^ are statistically about twice as large as those based on *F*, and *R*- factors based on ALL data will be even larger.

### Fractional atomic coordinates and isotropic or equivalent isotropic displacement parameters (Å^2^)

**Table:**

	*x*	*y*	*z*	*U*_iso_*/*U*_eq_	
O1W	0.0457 (4)	0.2431 (10)	0.3624 (6)	0.193 (3)	
H10	0.0696	0.2068	0.3324	0.232*	
H20	0.0081	0.1989	0.3326	0.232*	
S1	1.01347 (6)	0.9344 (2)	0.17559 (9)	0.0605 (4)	
S2	1.09337 (7)	0.5527 (2)	0.56716 (9)	0.0720 (5)	
O1	0.87014 (12)	0.9697 (4)	0.28332 (19)	0.0433 (7)	
O2	0.79211 (13)	1.1140 (5)	0.37749 (19)	0.0493 (8)	
O3	0.69458 (16)	1.2284 (7)	0.3502 (3)	0.0796 (12)	
O4	0.69663 (12)	0.9277 (5)	0.18084 (19)	0.0467 (7)	
O5	0.67103 (18)	0.7887 (8)	0.2901 (3)	0.0880 (14)	
O6	0.73828 (13)	0.5785 (4)	0.14481 (18)	0.0455 (7)	
O7	0.71021 (18)	0.5906 (6)	−0.0050 (2)	0.0732 (11)	
O8	0.86480 (13)	0.5944 (4)	0.12682 (18)	0.0464 (7)	
O9	0.9131 (2)	0.3601 (6)	0.2159 (3)	0.0902 (14)	
N1	0.95480 (15)	0.7892 (5)	0.2819 (2)	0.0427 (9)	
H1A	0.9581	0.7239	0.3287	0.051*	
N2	1.06247 (16)	0.7629 (6)	0.3289 (2)	0.0490 (10)	
H2A	1.0987	0.7961	0.3256	0.059*	
N3	1.06233 (17)	0.6483 (6)	0.3986 (2)	0.0496 (10)	
H3A	1.0510	0.5342	0.3874	0.060*	
N4	1.0852 (2)	0.8905 (7)	0.4975 (3)	0.0695 (13)	
H4B	1.0779	0.9645	0.4526	0.083*	
H4C	1.0960	0.9345	0.5510	0.083*	
C1	0.89174 (18)	0.8500 (6)	0.2278 (3)	0.0396 (10)	
H1B	0.8940	0.9195	0.1759	0.047*	
C2	0.84790 (19)	0.6840 (6)	0.1959 (3)	0.0387 (10)	
H2B	0.8538	0.5977	0.2459	0.046*	
C3	0.77758 (18)	0.7409 (6)	0.1562 (3)	0.0387 (10)	
H3B	0.7693	0.8028	0.0983	0.046*	
C4	0.76213 (18)	0.8694 (6)	0.2203 (3)	0.0396 (10)	
H4A	0.7681	0.8044	0.2768	0.047*	
C5	0.8067 (2)	1.0374 (6)	0.2378 (3)	0.0423 (10)	
H5A	0.8048	1.0902	0.1800	0.051*	
C6	0.7926 (2)	1.1884 (7)	0.2936 (3)	0.0487 (11)	
H6A	0.8249	1.2848	0.3050	0.058*	
H6B	0.7514	1.2436	0.2612	0.058*	
C7	0.7401 (3)	1.1452 (8)	0.3977 (4)	0.0586 (13)	
C8	0.7464 (4)	1.0653 (12)	0.4877 (5)	0.102 (2)	
H8A	0.7080	1.0891	0.5004	0.153*	
H8B	0.7820	1.1220	0.5331	0.153*	
H8C	0.7532	0.9337	0.4872	0.153*	
C9	0.6553 (2)	0.8752 (8)	0.2213 (4)	0.0534 (12)	
C10	0.5887 (2)	0.9374 (11)	0.1689 (4)	0.0745 (16)	
H10A	0.5601	0.8955	0.1984	0.112*	
H10B	0.5758	0.8859	0.1096	0.112*	
H10C	0.5874	1.0705	0.1653	0.112*	
C11	0.7051 (2)	0.5239 (7)	0.0609 (3)	0.0499 (12)	
C12	0.6616 (3)	0.3661 (9)	0.0615 (4)	0.0735 (17)	
H12A	0.6389	0.3273	0.0011	0.110*	
H12B	0.6316	0.4054	0.0892	0.110*	
H12C	0.6864	0.2642	0.0948	0.110*	
C13	0.8985 (2)	0.4365 (7)	0.1448 (3)	0.0495 (11)	
C14	0.9150 (2)	0.3741 (8)	0.0673 (4)	0.0643 (14)	
H14A	0.9387	0.2602	0.0819	0.096*	
H14B	0.9405	0.4672	0.0523	0.096*	
H14C	0.8765	0.3543	0.0170	0.096*	
C15	1.00857 (19)	0.8254 (6)	0.2658 (3)	0.0418 (10)	
C16	1.0796 (2)	0.7105 (7)	0.4844 (3)	0.0495 (12)	

### Atomic displacement parameters (Å^2^)

**Table:**

	*U*^11^	*U*^22^	*U*^33^	*U*^12^	*U*^13^	*U*^23^
O1W	0.265 (7)	0.099 (4)	0.166 (5)	0.007 (5)	0.012 (5)	−0.014 (4)
S1	0.0520 (7)	0.0731 (9)	0.0629 (8)	0.0127 (7)	0.0280 (6)	0.0215 (7)
S2	0.0840 (10)	0.0872 (11)	0.0403 (7)	0.0242 (8)	0.0154 (6)	0.0036 (7)
O1	0.0333 (14)	0.0484 (18)	0.0460 (17)	−0.0008 (13)	0.0109 (13)	−0.0055 (14)
O2	0.0468 (17)	0.053 (2)	0.0451 (18)	0.0115 (15)	0.0115 (14)	−0.0022 (15)
O3	0.050 (2)	0.104 (3)	0.088 (3)	0.024 (2)	0.029 (2)	0.003 (3)
O4	0.0344 (14)	0.0564 (19)	0.0471 (17)	0.0005 (15)	0.0112 (13)	−0.0011 (16)
O5	0.062 (2)	0.121 (4)	0.089 (3)	−0.011 (2)	0.036 (2)	0.022 (3)
O6	0.0445 (16)	0.0522 (19)	0.0344 (15)	−0.0082 (15)	0.0066 (13)	−0.0036 (14)
O7	0.092 (3)	0.079 (3)	0.0369 (19)	−0.017 (2)	0.0069 (18)	−0.0039 (19)
O8	0.0526 (17)	0.0488 (18)	0.0352 (15)	0.0105 (16)	0.0118 (13)	−0.0008 (14)
O9	0.132 (4)	0.073 (3)	0.081 (3)	0.047 (3)	0.056 (3)	0.023 (2)
N1	0.0352 (18)	0.051 (2)	0.041 (2)	0.0027 (17)	0.0117 (15)	0.0089 (17)
N2	0.0342 (19)	0.069 (3)	0.044 (2)	0.0034 (18)	0.0148 (17)	0.008 (2)
N3	0.046 (2)	0.052 (2)	0.040 (2)	0.0041 (18)	0.0019 (17)	−0.0040 (18)
N4	0.079 (3)	0.073 (3)	0.051 (3)	−0.007 (2)	0.016 (2)	−0.015 (2)
C1	0.038 (2)	0.042 (2)	0.039 (2)	0.0045 (19)	0.0128 (18)	0.0030 (19)
C2	0.041 (2)	0.047 (2)	0.028 (2)	0.0074 (19)	0.0111 (18)	0.0022 (18)
C3	0.036 (2)	0.044 (2)	0.033 (2)	−0.001 (2)	0.0078 (17)	0.0021 (19)
C4	0.031 (2)	0.051 (3)	0.035 (2)	0.0046 (19)	0.0101 (17)	0.0036 (19)
C5	0.042 (2)	0.042 (2)	0.041 (2)	0.001 (2)	0.0108 (19)	0.000 (2)
C6	0.049 (3)	0.041 (3)	0.056 (3)	−0.004 (2)	0.017 (2)	−0.002 (2)
C7	0.059 (3)	0.057 (3)	0.065 (3)	0.003 (3)	0.028 (3)	−0.008 (3)
C8	0.145 (6)	0.096 (5)	0.087 (5)	0.033 (5)	0.068 (4)	0.017 (4)
C9	0.044 (3)	0.060 (3)	0.059 (3)	−0.012 (2)	0.021 (2)	−0.012 (3)
C10	0.041 (3)	0.103 (5)	0.080 (4)	−0.006 (3)	0.022 (3)	−0.016 (4)
C11	0.046 (3)	0.052 (3)	0.045 (3)	0.003 (2)	0.007 (2)	−0.009 (2)
C12	0.068 (3)	0.075 (4)	0.062 (3)	−0.022 (3)	0.003 (3)	−0.018 (3)
C13	0.054 (3)	0.046 (3)	0.047 (3)	0.006 (2)	0.016 (2)	0.006 (3)
C14	0.062 (3)	0.067 (4)	0.070 (3)	0.014 (3)	0.031 (3)	−0.008 (3)
C15	0.037 (2)	0.045 (3)	0.043 (2)	0.007 (2)	0.0136 (19)	−0.005 (2)
C16	0.034 (2)	0.066 (3)	0.045 (3)	0.009 (2)	0.010 (2)	−0.011 (2)

### Geometric parameters (Å, °)

**Table:**

O1W—H10	0.868 (10)	C1—C2	1.516 (6)
O1W—H20	0.867 (8)	C1—H1B	0.9800
S1—C15	1.669 (5)	C2—C3	1.530 (5)
S2—C16	1.684 (5)	C2—H2B	0.9800
O1—C1	1.430 (5)	C3—C4	1.501 (6)
O1—C5	1.434 (5)	C3—H3B	0.9800
O2—C7	1.324 (6)	C4—C5	1.528 (6)
O2—C6	1.439 (6)	C4—H4A	0.9800
O3—C7	1.196 (6)	C5—C6	1.503 (6)
O4—C9	1.345 (6)	C5—H5A	0.9800
O4—C4	1.439 (5)	C6—H6A	0.9700
O5—C9	1.200 (6)	C6—H6B	0.9700
O6—C11	1.342 (5)	C7—C8	1.501 (9)
O6—C3	1.435 (5)	C8—H8A	0.9600
O7—C11	1.192 (6)	C8—H8B	0.9600
O8—C13	1.338 (6)	C8—H8C	0.9600
O8—C2	1.430 (5)	C9—C10	1.500 (7)
O9—C13	1.195 (6)	C10—H10A	0.9600
N1—C15	1.335 (5)	C10—H10B	0.9600
N1—C1	1.440 (5)	C10—H10C	0.9600
N1—H1A	0.8600	C11—C12	1.496 (8)
N2—C15	1.349 (5)	C12—H12A	0.9600
N2—N3	1.382 (5)	C12—H12B	0.9600
N2—H2A	0.8600	C12—H12C	0.9600
N3—C16	1.358 (6)	C13—C14	1.471 (7)
N3—H3A	0.8600	C14—H14A	0.9600
N4—C16	1.312 (7)	C14—H14B	0.9600
N4—H4B	0.8600	C14—H14C	0.9600
N4—H4C	0.8600		
			
H10—O1W—H20	104.6 (8)	O2—C6—C5	110.3 (4)
C1—O1—C5	112.1 (3)	O2—C6—H6A	109.6
C7—O2—C6	116.6 (4)	C5—C6—H6A	109.6
C9—O4—C4	117.9 (4)	O2—C6—H6B	109.6
C11—O6—C3	117.9 (3)	C5—C6—H6B	109.6
C13—O8—C2	119.8 (3)	H6A—C6—H6B	108.1
C15—N1—C1	125.6 (4)	O3—C7—O2	123.8 (5)
C15—N1—H1A	117.2	O3—C7—C8	124.9 (5)
C1—N1—H1A	117.2	O2—C7—C8	111.3 (5)
C15—N2—N3	123.2 (4)	C7—C8—H8A	109.5
C15—N2—H2A	118.4	C7—C8—H8B	109.5
N3—N2—H2A	118.4	H8A—C8—H8B	109.5
C16—N3—N2	121.9 (4)	C7—C8—H8C	109.5
C16—N3—H3A	119.0	H8A—C8—H8C	109.5
N2—N3—H3A	119.0	H8B—C8—H8C	109.5
C16—N4—H4B	120.0	O5—C9—O4	123.2 (5)
C16—N4—H4C	120.0	O5—C9—C10	125.6 (5)
H4B—N4—H4C	120.0	O4—C9—C10	111.2 (5)
O1—C1—N1	106.4 (3)	C9—C10—H10A	109.5
O1—C1—C2	111.5 (3)	C9—C10—H10B	109.5
N1—C1—C2	110.1 (4)	H10A—C10—H10B	109.5
O1—C1—H1B	109.6	C9—C10—H10C	109.5
N1—C1—H1B	109.6	H10A—C10—H10C	109.5
C2—C1—H1B	109.6	H10B—C10—H10C	109.5
O8—C2—C1	107.6 (3)	O7—C11—O6	124.5 (4)
O8—C2—C3	107.9 (3)	O7—C11—C12	124.8 (5)
C1—C2—C3	112.2 (3)	O6—C11—C12	110.7 (4)
O8—C2—H2B	109.7	C11—C12—H12A	109.5
C1—C2—H2B	109.7	C11—C12—H12B	109.5
C3—C2—H2B	109.7	H12A—C12—H12B	109.5
O6—C3—C4	108.4 (3)	C11—C12—H12C	109.5
O6—C3—C2	109.1 (3)	H12A—C12—H12C	109.5
C4—C3—C2	109.1 (3)	H12B—C12—H12C	109.5
O6—C3—H3B	110.1	O9—C13—O8	122.9 (4)
C4—C3—H3B	110.1	O9—C13—C14	125.8 (5)
C2—C3—H3B	110.1	O8—C13—C14	111.4 (4)
O4—C4—C3	108.7 (3)	C13—C14—H14A	109.5
O4—C4—C5	110.3 (3)	C13—C14—H14B	109.5
C3—C4—C5	109.0 (3)	H14A—C14—H14B	109.5
O4—C4—H4A	109.7	C13—C14—H14C	109.5
C3—C4—H4A	109.7	H14A—C14—H14C	109.5
C5—C4—H4A	109.7	H14B—C14—H14C	109.5
O1—C5—C6	108.5 (3)	N1—C15—N2	114.9 (4)
O1—C5—C4	106.8 (3)	N1—C15—S1	125.8 (3)
C6—C5—C4	115.2 (4)	N2—C15—S1	119.3 (3)
O1—C5—H5A	108.7	N4—C16—N3	117.6 (5)
C6—C5—H5A	108.7	N4—C16—S2	124.1 (4)
C4—C5—H5A	108.7	N3—C16—S2	118.2 (4)
			
C15—N2—N3—C16	−107.9 (5)	C1—O1—C5—C6	−169.4 (3)
C5—O1—C1—N1	−178.7 (3)	C1—O1—C5—C4	65.9 (4)
C5—O1—C1—C2	−58.6 (4)	O4—C4—C5—O1	175.5 (3)
C15—N1—C1—O1	−116.8 (4)	C3—C4—C5—O1	−65.3 (4)
C15—N1—C1—C2	122.3 (5)	O4—C4—C5—C6	54.9 (5)
C13—O8—C2—C1	103.9 (4)	C3—C4—C5—C6	174.1 (4)
C13—O8—C2—C3	−134.9 (4)	C7—O2—C6—C5	−125.6 (4)
O1—C1—C2—O8	168.0 (3)	O1—C5—C6—O2	−64.5 (4)
N1—C1—C2—O8	−74.2 (4)	C4—C5—C6—O2	55.1 (5)
O1—C1—C2—C3	49.5 (4)	C6—O2—C7—O3	0.8 (7)
N1—C1—C2—C3	167.3 (3)	C6—O2—C7—C8	−178.4 (5)
C11—O6—C3—C4	129.2 (4)	C4—O4—C9—O5	2.9 (7)
C11—O6—C3—C2	−112.1 (4)	C4—O4—C9—C10	−176.6 (4)
O8—C2—C3—O6	73.5 (4)	C3—O6—C11—O7	7.1 (7)
C1—C2—C3—O6	−168.1 (3)	C3—O6—C11—C12	−173.9 (4)
O8—C2—C3—C4	−168.2 (3)	C2—O8—C13—O9	3.6 (7)
C1—C2—C3—C4	−49.9 (4)	C2—O8—C13—C14	−175.8 (4)
C9—O4—C4—C3	115.1 (4)	C1—N1—C15—N2	176.3 (4)
C9—O4—C4—C5	−125.6 (4)	C1—N1—C15—S1	−5.0 (7)
O6—C3—C4—O4	−63.5 (4)	N3—N2—C15—N1	8.6 (6)
C2—C3—C4—O4	177.8 (3)	N3—N2—C15—S1	−170.2 (3)
O6—C3—C4—C5	176.4 (3)	N2—N3—C16—N4	12.4 (7)
C2—C3—C4—C5	57.7 (4)	N2—N3—C16—S2	−166.9 (3)

### Hydrogen-bond geometry (Å, °)

**Table:**

*D*—H···*A*	*D*—H	H···*A*	*D*···*A*	*D*—H···*A*
O1W—H10···O5^i^	0.868 (10)	2.637 (4)	3.382 (11)	144.5 (5)
O1W—H20···O9^ii^	0.867 (8)	2.563 (4)	3.181 (9)	129.1 (5)
N1—H1A···S2^iii^	0.86	2.62	3.400 (4)	151
N2—H2A···O3^iv^	0.86	2.09	2.856 (5)	147
N3—H3A···O1W^v^	0.86	2.13	2.973 (9)	167
N4—H4B···O1W^vi^	0.86	2.43	3.244 (9)	159
N4—H4C···O1^iii^	0.86	2.49	3.323 (5)	164

Symmetry codes: (i) *x*−1/2, *y*−1/2, *z*; (ii) *x*−1, *y*, *z*; (iii) −*x*+2, *y*, −*z*+1; (iv) *x*+1/2, *y*−1/2, *z*; (v) *x*+1, *y*, *z*; (vi) *x*+1, *y*+1, *z*.
